# Screening of Tnfaip1-Interacting Proteins in Zebrafish Embryonic cDNA Libraries Using a Yeast Two-Hybrid System

**DOI:** 10.3390/cimb45100518

**Published:** 2023-10-10

**Authors:** Shulan Huang, Hongning Zhang, Wen Chen, Jiawei Wang, Zhen Wu, Meiqi He, Jian Zhang, Xiang Hu, Shuanglin Xiang

**Affiliations:** 1 State Key Laboratory of Developmental Biology of Freshwater Fish, College of Life Sciences, Hunan Normal University, Changsha 410081, China; huangshulan5920@163.com (S.H.); zhn@hunnu.edu.cn (H.Z.); chenwen@biochen.org (W.C.); wjw1079444680@163.com (J.W.); 13100311591@163.com (Z.W.); hemeiqi75@163.com (M.H.); zhangjian@hunnu.edu.cn (J.Z.); 2 Engineering Research Center for Antibodies from Experimental Animals of Hunan Province, College of Life Sciences, Hunan Normal University, Changsha 410081, China

**Keywords:** Tnfaip1, zebrafish embryos, cDNA library, yeast two-hybrid system, interacting protein

## Abstract

TNFAIP1 regulates cellular biological functions, including DNA replication, DNA repair, and cell cycle, by binding to target proteins. Identification of Tnfaip1-interacting proteins contributes to the understanding of the molecular regulatory mechanisms of their biological functions. In this study, 48 hpf, 72 hpf, and 96 hpf wild-type zebrafish embryo mRNAs were used to construct yeast cDNA library. The library titer was 1.12 × 10^7^ CFU/mL, the recombination rate was 100%, and the average length of the inserted fragments was greater than 1000 bp. A total of 43 potential interacting proteins of Tnfaip1 were identified using zebrafish Tnfaip1 as a bait protein. Utilizing GO functional annotation and KEGG signaling pathway analysis, we found that these interacting proteins are mainly involved in translation, protein catabolic process, ribosome assembly, cytoskeleton formation, amino acid metabolism, and PPAR signaling pathway. Further yeast spotting analyses identified four interacting proteins of Tnfaip1, namely, Ubxn7, Tubb4b, Rpl10, and Ybx1. The Tnfaip1-interacting proteins, screened from zebrafish embryo cDNA in this study, increased our understanding of the network of Tnfaip1-interacting proteins during the earliest embryo development and provided a molecular foundation for the future exploration of tnfaip1’s biological functions.

## 1. Introduction

TNF alpha-induced protein 1 (TNFAIP1) was first identified in human umbilical vein endothelial cells and can be induced by tumor necrosis factor α (TNFα) [1]. TNFAIP1 is a member of the PDIP1 subfamily of the KCTD protein family, and it functions through protein–protein interactions to form homomers or heterodimers [2]. This protein family also includes two members, KCTD10 and KCTD13. Studies have shown that KCTD10 is involved in early cardiac morphogenesis and vascular development [3,4,5]; KCTD13 is involved in the regulation of early embryonic neurodevelopment [6,7,8] and is considered to be an important neurodevelopmental regulator. TNFAIP1 interacts with Rnd2 and Rnd3 to regulate the lateral migration of neuronal cells and influence the dynamics of neuronal branching and dendritic spines, suggesting the role of TNFAIP1 in early embryonic neural development [9,10]. These findings suggest that members of the PDIP1 protein family have important regulatory roles during early embryonic development. TNFAIP1 is highly conserved among different species and contains an N-terminal BTB/POZ domain and a C-terminal PCNA-binding motif “QTKVEFP” [11]. Studies have shown that TNFAIP1 regulates tumors progression [12,13] and affects the development and differentiation of neurons in the embryonic cerebral cortex [9,10] through the interaction with target proteins. Zebrafish *tnfaip1* is located on chromosome 15 and encodes a protein of 320 amino acids with a molecular weight of 36.8 KD. Our previous findings indicate that *tnfaip1* is highly expressed during early embryonic development, and the inhibition of *tnfaip1* expression affects eye and head development in zebrafish embryos [14], but the specific regulatory mechanisms are unclear. Zebrafish (*Danio rerio*) is highly homologous to the human genome sequence [15] and is very similar in organ composition, physiological functions, and developmental processes, and it has been widely used in studies of early developmental regulation. Therefore, in the present study, we constructed a yeast cDNA library using zebrafish embryos, in order to screen out the interacting proteins of Tnfaip1 during early embryonic development.

Yeast two-hybrid technology is a highly sensitive assay for discovering new protein–protein interactions [16]. The principle is to use the properties of the DNA-binding domain (DNA-BD) and the transcriptional activation domain (DNA-AD) of the eukaryotic transcription factor Gal4. The bait gene and target protein DNA library were ligated to the BD and AD domains, respectively, and co-transformed into yeast competent cells. If there is an interaction between the bait and the target protein, the BD and AD domains of the transcription factor Gal4 are spatially close enough to activate the expression of the downstream reporter genes as a way of identifying the two proteins that are interacting [17,18]. The technique is simple and sensitive and can realistically reflect protein–protein interactions in living cells. Many biological processes in organisms are co-regulated by protein complexes, and the formation of which is based on protein–protein interactions. The identification of interacting proteins of Tnfaip1 is an important strategy for understanding *tnfaip1* function. Therefore, in the present study, the yeast two-hybrid system was performed to screen the proteins interacting with Tnfaip1 in early embryonic development using zebrafish tnfaip1 as a bait, and the cDNA of zebrafish early embryos was used as a screening library to provide an important molecular basis for elucidating the biological functions of *tnfaip1*.

In this study, we constructed a yeast two-hybrid cDNA library from 48 hpf, 72 hpf, and 96 hpf embryos of zebrafish, and we screened 43 potential interacting proteins using Tnfaip1 as a bait protein. Functional annotation of these potential interacting target proteins further explained the potential biological regulatory functions of *tnfaip1*. These findings increase our understanding of the Tnfaip1-interacting protein network and provide an important molecular basis for future exploration of the function of Tnfaip1 in early embryonic development.

## 2. Materials and Methods

### 2.1. *Materials and Reagents*

Zebrafish (TU strain) were obtained from the China Zebrafish Resource Center (Wuhan, China). Zebrafish embryo cDNA library, Y2HGold Yeast Strain, pGADT7 Vector, pGBKT7 Vector, pGADT7-T Vector, pGBKT7-53 Vector, and pGBKT7-Lam Vector were purchased from Zoonbio Biotechnology (Nanjing, China). T4 DNA ligase and restriction endonuclease were purchased from Thermo Fisher Scientific (Waltham, MA, USA). *E.coli* top 10 was prepared and preserved in the laboratory. Yeast complete medium and nutrient-deficient medium were purchased from Coolaber (Beijing, China). Carrier DNA, 3-Amino-1,2,4-triazole (3-AT), and 5-Bromo-4-chloro-3-indolyl-α-D-galactopy-ranoside (X-α-Gal) were purchased from Sigma (St. Louis, MO, USA). PCR mix, DNA marker, and agarose gel purification and recovery kit were purchased from Vazyme (Nanjing, China). The yeast plasmid extraction kit was purchased from Solarbio (Beijing, China). The gene sequences and primers were synthesized by Tsingke (Beijing, China).

### 2.2. Zebrafish Husbandry

Adult zebrafish were bred under 14 h/10 h day/night cycle at 28.5 °C. Adult male and female zebrafish were mated in a 1:1 ratio to obtain embryos and staged in hour post-fertilization (hpf). Fertilized eggs were cultured at 28.5 °C in E3 water (5 mM NaCl, 0.17 mM KCl, 0.33 mM CaCl_2_·H_2_O, 0.33 mM MgSO_4_·7H_2_O).

### 2.3. Assays of Toxicity and Auto-Activation of the Zebrafish Tnfaip1 Bait Plasmid

The recombinant bait plasmid was constructed by cloning the CDS sequence of zebrafish *tnfaip1* into the pGBKT7 yeast vector using a homologous recombination technique. The recombinant bait plasmid pGBKT7-*tnfaip1* and pGADT7 prey empty vector were co-transformed into Y2H Gold yeast competent cells. The transformants were coated onto Minimal media Double Dropout (DDO, SD/-Trp/-Leu), Minimal media Triple Dropout (TDO, SD/-Trp/-Leu/-His), and Minimal media Quadruple Dropout (QDO, SD/-Trp/-Leu/-His/-Ade) plates, respectively, and incubated at 30 °C for 3–5 d. The growth status of the yeast clones was observed. pGBKT7-53 and pGADT7-T were co-transformed and coated onto DDO and QDO plates as positive controls; pGBKT7-lam and pGADT7-T were co-transformed and coated onto DDO and QDO plates as negative controls. If the bait plasmid grows on TDO and QDO plates, it means that the bait can activate the expression of HIS3 and ADE2, which indicates that the bait protein has auto-activation viability and should be inhibited of its auto-activation. The bait plasmid pGBKT7-*tnfaip1* and pGADT7 prey empty vector were co-transformed into Y2H Gold yeast competent cells and coated onto QDO plates with different concentrations of 3-Amino-1,2,4-triazole (3-AT), to determine the optimal concentration of 3-AT to inhibit the viability of the bait protein’s auto-activation for subsequent library screening.

### 2.4. Screening and Identification of Tnfaip1-Interacting Proteins

A total of 5 μg of pGBKT7-*tnfaip1* bait plasmid, 10 μg of zebrafish embryonic yeast library plasmid, and 50 μL of pre-denatured Carrier DNA were co-transformed into Y2H Gold yeast competent cells. A 3 mL volume of YPD Plus liquid medium was added and incubated at 30 °C for 90 min with shaking, and then centrifuged at 700× *g* for 5 min, and subsequently, the supernatant was discarded. Bacteria were resuspended in 6 mL of 0.9% NaCl solution. The bacterial solution was coated on QDO + 40 mM 3-AT plate at 150 μL/plate, and incubated at 30 °C for 3–5 d. The well-growing single clones on the primary screening plate were spotted onto QDO/X-α-Gal + 40 mM 3-AT plate, and incubated at 30 °C for 3–5 d. All clones that grew and turned blue during rescreening were amplified with colony PCR using the universal primers of the prey vector pGADT7, and the PCR products were sequenced. Sequencing results were analyzed using Blast with the NCBI database to identify positive cloned genes.

### 2.5. Functional Annotation of Potential Tnfaip1-Interacting Proteins

All positive cloned genes screened were enriched using GO with the “enrich GO” function and enriched using KEGG with the “enrich KEGG” function of cluster Profiler in the R package (https://www.r-project.org/ accessed on 8 October 2023), and the enrichment results were visualized with the ggplot2 package. *p*-adjust < 0.05 was considered significantly enriched.

### 2.6. Yeast Two-Hybrid Spot Analysis

Further point-to-point verification was performed on the following Tnfaip1-interacting proteins: Ubxn7 (NP_001001951.1), Tubb4b (NP_942104.2), Rpl10 (XP_017209012.1), and Ybx1 (AAH50156.1). These proteins were screened from the zebrafish embryonic yeast library. Plasmid extraction was performed according to the yeast plasmid extraction kit’s instructions. A 300 ng quantity of pGBKT7-*tnfaip1* bait plasmid and the corresponding prey plasmid were co-transformed into Y2H Gold yeast competent cells. The pGBKT7-53 + pGADT7-T was used as a positive control, pGBKT7-Lam + pGADT7-T as a negative control, pGBKT7-tnfaip1+ pGADT7 prey empty vector as an auto-activation control, and pGBKT7-tnfaip1+ positive clone plasmid as a co-transformation experimental group. The transformants were coated on DDO, TDO, and QDO plates and incubated at 30 °C for 3–5 d. A single clone grown on the QDO plate was picked, gradient diluted, and spotted onto DDO and QDO/X-α-Gal plates, incubated at 30 °C for 3–5 d, and its status of bacterial spot growth was observed.

## 3. Results

### 3.1. Construction of a Yeast Library of cDNA from Zebrafish Embryos

**Figure 1:**
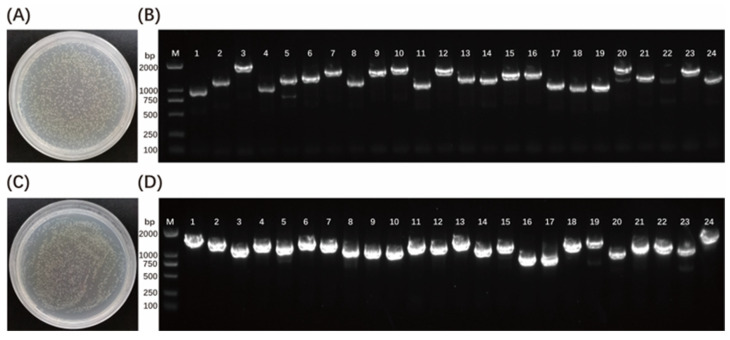
Construction of zebrafish embryo cDNA yeast library. (**A**,**C**) Library titer identification of primary and secondary library plasmids. (**B**,**D**) Primary and secondary library plasmid recombination rate and insert fragment length identification. M: DL2000 DNA marker. 1–24: 24 clones were randomly selected from the primary and secondary zebrafish embryo cDNA yeast two-hybrid libraries.

Wild-type zebrafish embryos developed up to 48 hpf, 72 hpf, and 96 hpf were collected for total RNA extraction and aliquot yeast cDNA library construction. Gateway recombination technology was used to construct zebrafish embryo cDNA yeast library plasmids. First, zebrafish embryo cDNA was cloned into the primary vector pDONR222 to obtain the primary library plasmid, with a library titer of 1.20 × 10^7^ CFU/mL (Figure 1A), and the average length of the insert fragments was longer than 1000 bp, and the recombination rate was 100% (Figure 1B). Then, the primary library plasmid was cloned into the pGADT7-DEST plasmid in a secondary recombination reaction to obtain a secondary cDNA library, with a library titer of 1.12 × 10^7^ CFU/mL (Figure 1C), and the average length of the insert fragments was longer than 1000 bp, and the recombination rate was 100% (Figure 1D). The results showed that the zebrafish embryonic yeast cDNA library was successfully constructed, and it met the library screening requirements for interacting proteins.

### 3.2. Toxicity and Auto-Activation Detection of Zebrafish Tnfaip1 Bait Recombinant Plasmid

**Figure 2:**
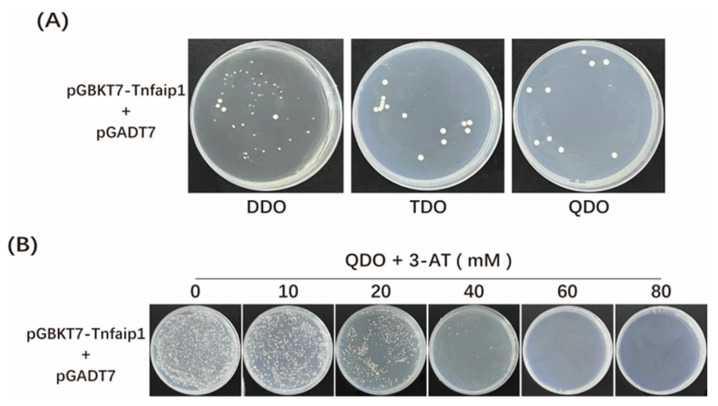
Detection of toxicity and auto-activation activity of bait plasmid pGBKT7-Tnfaip1. (**A**) Toxicity and auto-activation activity of bait plasmids in yeast cells. The bait plasmid pGBKT7-*tnfaip1* and the prey empty vector pGADT7 were co-transformed into yeast competent cells and coated onto DDO plates for toxicity assessment and coated onto TDO and QDO plates for auto-activation activity assays. (**B**) Concentration screening of 3-AT inhibition bait plasmid auto-activation activity. The bait plasmid pGBKT7-*tnfaip1* and the prey empty vector pGADT7 were co-transformed into yeast competent cells and coated onto QDO plates with different concentrations of 3-AT for the auto-activation inhibition assay. DDO: Minimal media Double Dropout plate, SD/-Trp/-Leu; TDO: Minimal media Triple Dropout plate, SD/-Trp/-Leu/-His; QDO: Minimal media Quadruple Dropouts, SD/-Trp/-Leu/-His/-Ade plate.

The bait plasmid pGBKT7-*tnfaip1* and pGADT7 prey empty vector were co-transformed into Y2H Gold yeast competent cells. Yeast cells grew on DDO plates, indicating that the bait plasmid pGBKT7-*tnfaip*1 was successfully transferred into the host bacteria and was not toxic to the host bacteria (Figure 2A). There was a little clone growth on TDO and QDO plates (Figure 2A), indicating that the bait activates the expression of HIS3 and ADE2, suggesting that the bait proteins have a slight auto-activation viability and need to be subjected to auto-activation inhibition. The bait plasmid pGBKT7-*tnfaip*1 and pGADT7 prey empty vector were co-transformed into Y2H Gold yeast competent cells and coated onto QDO plates supplemented with 0–80 mM 3-AT for the auto-activation inhibition assay. Transformants showed no significant growth on plates of QDO + 40 mM 3-AT (Figure 2B); therefore, this concentration was used for subsequent library screening experiments.

### 3.3. Screening and Sequencing Analysis of Tnfaip1-Interacting Proteins

**Figure 3:**
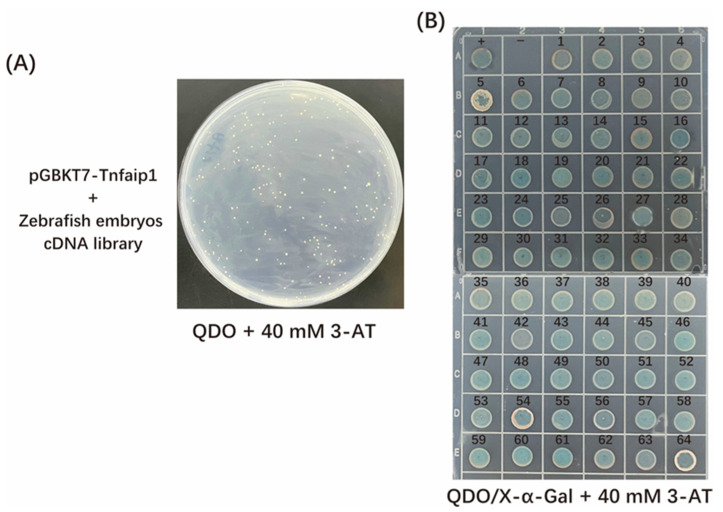
Screening of zebrafish Tnfaip1-interacting proteins. (**A**) Tnfaip1-interacting protein primary screening’s plate clone growth. The bait plasmid pGBKT7-tnfaip1 and the zebrafish embryo yeast cDNA library plasmid were co-transformed into Y2H Gold yeast competent cells and coated on QDO + 40 mM 3-AT plates for positive interacting protein screening. (**B**) Tnfaip1-interacting protein spot plate rescreening of positive clones. The well-growing single clones were spotted onto QDO/X-α-Gal + 40 mM 3-AT plates for secondary screening. +: positive control; −: negative control; 1–64: positive clones containing Tnfaip1-interacting proteins.

The pGBKT7-*tnfaip1* bait plasmid and zebrafish embryonic yeast cDNA library plasmid were co-transformed into Y2H Gold yeast competent cells, coated on QDO + 40 mM 3-AT plates, and cultured at 30 °C for 3–5 d (Figure 3A). The 64 clones with excellent growth on the primary screening plate were spotted onto QDO/X-α-Gal + 40 mM 3-AT plate and cultured at 30 °C for 3–5 d for secondary screening to remove fake positive clones (Figure 3B). The positive clones that grew on the spot plate and turned blue were amplified using colony PCR with the universal primers of the prey vector pGADT7, and the PCR amplified products were sent for sequencing. The sequencing results were subjected to Blast analysis in the NCBI database, which removed repeat clones and empty vectors, and eventually, 43 Tnfaip1-interacting proteins were obtained. These proteins included ubiquitin-related proteins, ribosomal proteins, transcription factors, cytoskeleton proteins, proteasome-related proteins, etc. (Table 1).

### 3.4. Functional Enrichment Analysis of Tnfaip1-Interacting Proteins

**Figure 4:**
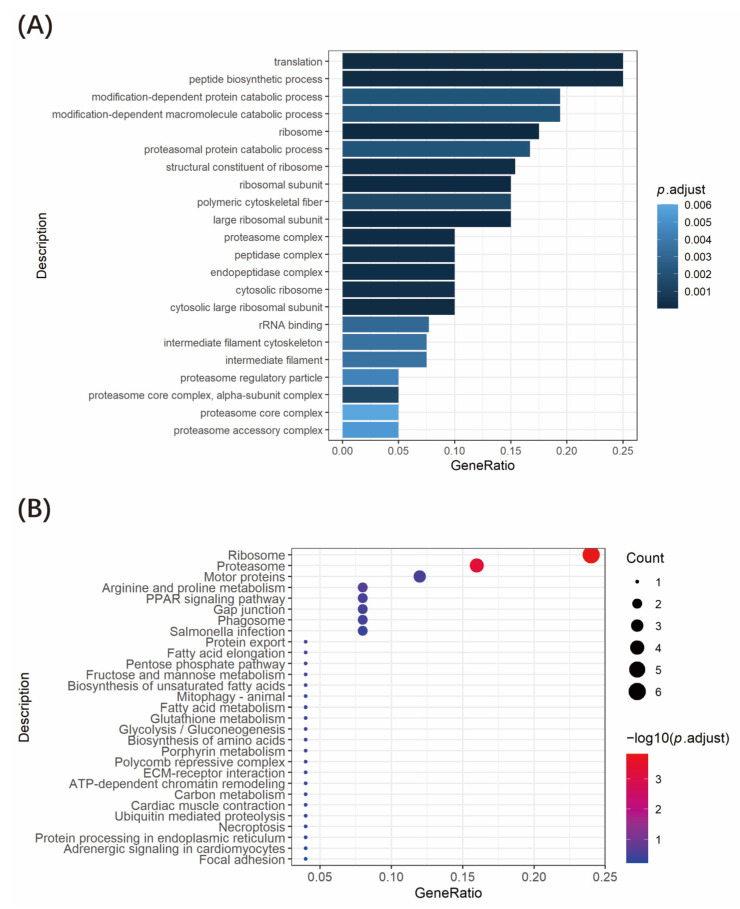
GO and KEGG signaling pathway enrichment analysis of zebrafish Tnfaip1-interacting protein. (**A**) GO functional annotation of Tnfaip1-interacting proteins. (**B**) KEGG signaling pathway enrichment analysis of Tnfaip1-interacting proteins.

We performed GO functional annotation and KEGG signaling pathway enrichment analysis on the 43 Tnfaip1-interacting proteins screened above. GO functional annotation indicates that these genes are mainly involved in the regulation of translation, protein catabolic process, ribosome assembly, cytoskeletal fiber formation, etc. (Figure 4A). KEGG signaling pathway enrichment analysis suggested that these genes are involved in ribosome, proteasome, motor proteins, arginine and proline metabolism, PPAR signaling pathway, etc. (Figure 4B). Taken together, zebrafish Tnfaip1-interacting proteins are involved in a variety of biological regulatory processes, suggesting that Tnfaip1 plays an important role in early embryonic development through its interaction with these proteins.

### 3.5. Yeast Spotting Analysis of the Interaction between Tnfaip1 and Ubxn7, Tubb4b, Rpl10, and Ybx1 

**Figure 5:**
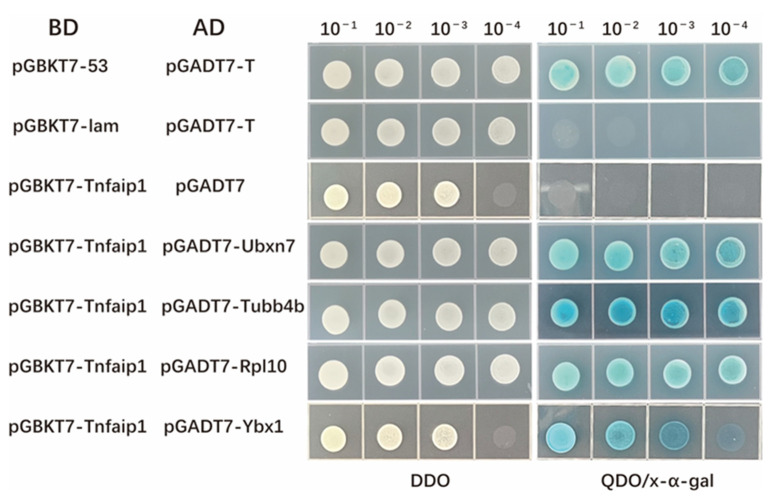
Yeast two-hybrid point-to-point validation of Tnfaip1-interacting proteins. The pGBKT7-*tnfaip1* bait plasmid and interacting protein Ubxn7, Tubb4b, Rpl10, and Ybx1 were co-transformed into Y2H Gold yeast competent cells, and their gradient dilution was spotted onto DDO and QDO/X-α-Gal plates. Positive control: pGBKT7-53 + pGADT7-T; negative control: pGBKT7-Lam + pGADT7-T, pGBKT7-Tnfaip1 + pGADT7 prey empty vector.

To further validate the interaction of Tnfaip1 with these potential target proteins. The positive clones Ubxn7 (NP_001001951.1), Tubb4b (NP_942104.2), Rpl10 (XP_017209012.1), and Ybx1 (AAH50156.1) prey plasmids were subjected to yeast plasmid extraction based on the results of functional enrichment analysis. The pGBKT7-*tnfaip1* bait plasmid and positive cloned plasmid DNA were co-transformed into Y2H Gold yeast competent cells for point-to-point validation. Their gradient dilution was spotted onto DDO and QDO/X-α-Gal plates using pGBKT7-53 + pGADT7-T as a positive control, pGBKT7-Lam + pGADT7-T as a negative control, and pGBKT7-tnfaip1+ pGADT7 prey empty vector as a auto-activation control. All spot plate combinations grew normally on DDO plates, positive controls grew and turned blue on QDO/X-α-Gal plates, negative controls did not grow on QDO/X-α-Gal plates, and Tnfaip1 co-transformed with Ubxn7, Tubb4b, Rpl10, and Ybx1 prey plasmids grew and turned blue on QDO/X-α-Gal plates (Figure 5). These results suggested an interaction between Tnfaip1 and Ubxn7, Tubb4b, Rpl10, and Ybx1.

## 4. Discussion

Embryonic development refers to the process of cell growth, differentiation, and morphogenesis that allow organisms to form tissues and organs, and it is accurately regulated by gene expression patterns. Zebrafish (*Danio rerio*) are highly homologous with the human genome sequence [15] and is very similar in organ composition, physiological functions, and developmental processes. As an important vertebrate model organism, zebrafish is widely used in the fields of early developmental regulation, study of gene function, establishment of animal models of human diseases [19], screening of clinical drugs [20], and epigenetics [21]. TNFAIP1 is a member of the PDIP1 subfamily of the KCTD protein family, and it functions through protein–protein interaction to form homomers or heterodimers [2]. This protein family also includes two members, KCTD10 and KCTD13. Studies have shown that KCTD10 is involved in early cardiac morphogenesis and vascular development [3,4,5]; KCTD13 is involved in the regulation of early embryonic neurodevelopment [6,7,8] and is considered to be an important neurodevelopmental regulator. TNFAIP1 interacts with Rnd2 and Rnd3 to regulate the lateral migration of neuronal cells and influence the dynamics of neuronal branching and dendritic spines, suggesting the role of TNFAIP1 in early embryonic neural development [9,10]. These findings suggest that members of the PDIP1 protein family have important regulatory roles during early embryonic development. Studies have shown that TNFAIP1 mediates DNA replication, repair, and cell cycle regulation by interacting with PCNA [11], and it regulates hepatocellular carcinoma progression by interacting with CSNK2B [13]. Our previous results indicate that Tnfaip1 is highly expressed during early development of zebrafish embryos, and the inhibition of Tnfaip1 expression affects the size of the eyes and head of zebrafish embryos [14], further suggesting a role of Tnfaip1 in early embryonic development. The biological functions of TNFAIP1 have been gradually elucidated, and its specific molecular regulatory mechanisms need to be further elucidated. Therefore, we hoped to identify its molecular regulatory network by screening Tnfaip1-interacting proteins on a large scale.

With the emergence of the yeast two-hybrid system and its continuous improvement, the system has developed into a mature tool for protein–protein interaction research [17,22,23]. The technique is simple and sensitive and can realistically reflect protein–protein interactions in living cells. At present, it is widely used in the screening of interacting proteins [18], the drawing of protein linkage maps [24], drug discovery [25], and the exploration of plant cell signaling pathways [26].

High-quality cDNA library is the experimental basis for screening interacting proteins in the yeast two-hybrid system. In this study, the Gateway recombination technology was used to construct a zebrafish embryonic yeast cDNA library. First, zebrafish embryo cDNA was cloned into the primary vector pDONR222 to obtain the primary library plasmid, with a library titer of 1.20 × 10^7^ CFU/mL, and the average length of the insert fragments was longer than 1000 bp, and the recombination rate was 100%. Then, the primary library plasmid was cloned into the pGADT7-DEST plasmid using a secondary recombination reaction to obtain a secondary cDNA library, with a library titer of 1.12 × 10^7^ CFU/mL, and the average length of the insert fragments was longer than 1000 bp, and the recombination rate was 100%. These results indicate that the library has enough coverage, high recombination rate, and high integrity to meet the library screening requirements of interacting proteins.

Species homology analysis revealed that the *TNFAIP1* gene is highly conserved among different species, with 73% sequence homology between zebrafish and humans. Zebrafish Tnfaip1 was used as a bait protein, and the *tnfaip1* protein coding sequence was cloned into the pGBKT7 yeast vector to construct a recombinant bait plasmid. After toxicity and auto-activation assays were performed, we found that the Tnfaip1 recombinant bait plasmid was not toxic to yeast cells but had a slight auto-activation activity. Thus, we chose 40 mM 3-AT as the growth stress for the auto-activation of the bait plasmid.. The pGBKT7-*tnfaip1* bait plasmid and zebrafish embryonic cDNA library plasmid were co-transformed into yeast competent cells. A total of 43 potential interacting proteins of Tnfaip1 were obtained by reporter gene testing as well as positive clone sequencing analysis. These proteins included ubiquitin-related proteins, ribosomal proteins, transcription factors, cytoskeleton proteins, proteasome-related proteins, etc. (Table 1). GO functional annotation and KEGG enrichment analysis of these potentially interacting proteins indicated that they are mainly involved in the regulation of translation, protein catabolic process, ribosome assembly, cytoskeleton formation, amino acid metabolism, and PPAR signaling pathway. The discovery of potential interacting target proteins of Tnfaip1 provides an important molecular basis for investigating its regulatory mechanisms during early embryonic development.

Further yeast spotting analysis confirmed the interactions between Tnfaip1 and Ubxn7, Tubb4b, Rpl10, and Ybx1. UBXN7 encodes a protein with ubiquitin-binding activity and ubiquitin-protein ligase-binding activity. Previous studies have confirmed the interaction between homo sapiens UBXN7 and TNFAIP1 [27]. UBXN7 as a ubiquitin ligase cofactor participates in the regulation of protein expression that affects developmental processes [28,29]. Tubb4b protein belongs to the β-tubulin protein family, which can affect cell proliferation and cell cycle, and it is essential for cell growth and development [30]. RPL10 is a ribosomal protein that is a component of the 60S ribosomal subunit. It was found that the variation of this gene leads to X-linked syndrome, which results in microcephaly and neurodevelopmental abnormalities [31,32]. Ybx1 is a DNA- and RNA-binding protein that regulates gene transcription, DNA repair and replication, RNA processing and stability, and protein translation [33,34,35], thereby affecting early embryonic development. Taken together, the interacting proteins of Tnfaip1 are related to early embryonic development, suggesting that the role of Tnfaip1 during early development may be a result of its binding to these target proteins.

## 5. Conclusions

In this study, we constructed a yeast two-hybrid cDNA library for zebrafish early embryos undergoing development. Using zebrafish Tnfaip1 as a bait protein, 43 potential interacting proteins were screened. According to functional annotation, these potential interacting proteins are mainly involved in translation, protein catabolic process, ribosome assembly, cytoskeleton formation, amino acid metabolism, and PPAR signaling pathway. Further yeast spotting analysis confirmed the interaction of Tnfaip1 with Ubxn7, Tubb4b, Rpl10, and Ybx1. The identification of Tnfaip1-interacting proteins involved in early embryonic development lays the molecular foundation for understanding the biological functions of *tnfaip1*.

## Figures and Tables

**Table 1:** Tnfaip1-interacting proteins identified in zebrafish embryo cDNA yeast library analyzed by Blast with the NCBI database.

**Clone** **No.**	**GenBank** **Accession No.**	**Gene Name**	**Identify**
1	NP_001001951.1	UBX domain protein 7 (ubxn7)	100%
3	NP_998223.1	heat shock protein 5 (hspa5)	98%
4	AAI63717.1	Laminin, alpha 4 (lama4)	100%
6	NP_942104.2	tubulin, beta 4B class IVb (tubb4b)	100%
7	NP_571519.1	activated C kinase 1 (rack1)	100%
8	AAH71464.1	eukaryotic translation elongation factor 1 beta 2(eef1b2)	99%
9	NP_571182.1	type I cytokeratin, enveloping layer (cyt1)	97%
10	NP_571180.1	alpha-tropomyosin (tpma)	100%
12	AAH45463.1	proteasome 26S subunit, non-ATPase, 1 (psmd1)	99%
16	NP_956269.1	uncharacterized protein zgc:65894 (zgc:65894)	99%
17	NP_705941.2	proteasome 20S subunit alpha 6a (psma6a)	99%
18	NP_001103596.2	ChaC, cation transport regulator homolog 1 (chac1)	99%
19	NP_937783.1	tumor protein, translationally-controlled 1 (tpt1)	99%
20	XP_005170972.1	myozenin 2b (myoz2b)	99%
21	XP_005157650.1	creatine kinase, muscle b (ckmb)	100%
22	AAH67580.1	ribosomal protein L4 (rpl4)	100%
23	XP_002662576.2	Mov10 RISC complex RNA helicase a (mov10a)	98%
24	NP_958456.1	trans-2,3-enoyl-CoA reductase b (tecrb)	99%
25	XP_017209012.1	ribosomal protein L10 (rpl10)	100%
26	NP_571694.1	myosin, light polypeptide 3, skeletal muscle (mylz3)	99%
27	AAH45406.1	actin, alpha 1, skeletal muscle	99%
29	NP_001003861.1	ribosomal protein L9 (rpl9)	99%
30	NP_956324.1	ribosomal protein L27a (rpl27a)	100%
32	AAH65432.1	ribosomal protein L8 (rpl8)	100%
33	AAQ91246.1	laminin receptor 1 (LAMR1)	99%
35	AAH44379.1	aldolase a, fructose-bisphosphate, a (aldoaa)	100%
36	AAH66728.1	keratin 4 (krt4)	99%
37	XP_021332536.1	matrilin 4 (matn4)	99%
38	AAH56706.1	creatine kinase, muscle a (ckma)	100%
39	NP_001077313.1	collagen, type XI, alpha 1a (col11a1a)	100%
41	NP_001373589.1	dual specificity phosphatase 27 (dusp27)	100%
42	XP_005173906.4	vimentin-like	99%
43	NP_001093614.2	apolipoprotein A-Ib (apoa1b)	97%
45	NP_001002498.2	holocytochrome c synthase a (hccsa.1)	98%
48	NP_001071272.2	ubiquitin C (ubc)	99%
50	AAH50156.1	Y box binding protein 1 (ybx1)	99%
53	NP_999862.1	proteasome 20S subunit alpha 4 (psma4)	98%
54	NP_001017824.1	keratin 92 (krt92)	99%
55	NP_001017766.2	embryonic ectoderm development (eed)	98%
57	NP_001002106.1	ribosomal protein L5b (rpl5b)	90%
60	NP_001036788.1	H2A.Z variant histone 2b (h2az2b)	99%
62	NP_938181.1	translocase of inner mitochondrial membrane 17 homolog A (timm17a)	98%
63	NP_956585.1	proteasome 26S subunit, non-ATPase 6 (psmd6)	99%

## Data Availability

Not applicable.
